# Meat consumption and the risk of hip fracture in women and men: two prospective Swedish cohort studies

**DOI:** 10.1007/s00394-024-03385-z

**Published:** 2024-04-17

**Authors:** Eva Warensjö Lemming, Liisa Byberg, Jonas Höijer, John A. Baron, Alicja Wolk, Karl Michaëlsson

**Affiliations:** 1 Medical Epidemiology, Department of Surgical Sciences, Uppsala University, Uppsala Science Park, MTC/Epihubben, Dag Hammarskjölds väg 14B, 751 83 Uppsala, Sweden; 2 Department of Food Studies, Nutrition and Dietetics, Uppsala University, Uppsala, Sweden; 3 Department of Medicine, University of North Carolina School of Medicine, Chapel Hill, NC USA; 4 Department of Epidemiology, Gillings School of Global Public Health, University of North Carolina, Chapel Hill, NC USA; 5 Unit of Cardiovascular and Nutritional Epidemiology, Institute of Environmental Medicine, Karolinska Institutet, Stockholm, Sweden

**Keywords:** Meat intake, Hip fracture, Biomarkers, Bone turnover, Cohort

## Abstract

**Purpose:**

To study the association between meat intake (predominantly red and processed meats) and the risk of hip fracture, as well as the association between meat intake and biomarkers of inflammation, oxidative stress, bone turnover, body composition, and bone mineral density (BMD).

**Methods:**

Data from the Swedish Mammography Cohort and the Cohort of Swedish men (n = 83,603, 54% men) with repeated investigations and their respective clinical sub-cohorts was utilised. Incident hip fractures were ascertained through individual linkage to registers. Associations were investigated using multivariable Cox and linear regression analyses.

**Results:**

During up to 23 years of follow-up (mean 18.2 years) and 1,538,627 person-years at risk, 7345 participants (2840 men) experienced a hip fracture. Each daily serving of meat intake conferred a hazard ratio (HR) of 1.03 (95% confidence interval [CI] 1.00; 1.06) for hip fracture. In quintile 5, compared to quintile 2, the HR was 1.11 (95% CI 1.01; 1.21) among all participants. In the sub-cohorts, meat intake was directly associated with circulating levels of interleukin-6, C-reactive protein, leptin, ferritin, parathyroid hormone, and calcium.

**Conclusion:**

A modest linear association was found between a higher meat intake and the risk of hip fractures. Our results from the sub-cohorts further suggest that possible mechanisms linking meat intake and hip fracture risk may be related to the regulation of bone turnover, subclinical inflammation, and oxidative stress. Although estimates are modest, limiting red and processed meat intake in a healthy diet is advisable to prevent hip fractures.

**Supplementary Information:**

The online version contains supplementary material available at 10.1007/s00394-024-03385-z.

## Background

Throughout life, the skeleton continuously attempts to renew and preserve bone quality through coupled bone turnover, which maintains calcium homeostasis and reduces bone brittleness. Nonetheless, bone mineral density (BMD) progressively declines with age, where the bone becomes more fragile, which leads to osteoporosis and a higher rate of fractures [1]. Fractures are common in elderly individuals [2], where the lifetime cumulative incidence is up to 50% in women and 25% in men [3, 4]. Hip fracture is the most devastating fragility fracture, leading to a high burden of morbidity and mortality and increased health care costs [5]. However, in addition to age-related bone loss [1], sarcopenia [6, 7] contributes to the fracture risk by affecting balance and causing falls. In addition to reduced muscle mass, sarcopenia leads to diminished muscle strength through loss of muscle fibers, fatty degeneration, fibrotic changes, and a reduced number of functioning neuromuscular units [8, 9]. The evidence shows that age-related bone loss and sarcopenia share pathogenic mechanisms such as oxidative stress and inflammation [1, 6, 7]. A healthy diet may benefit both, resulting in fewer fractures [10]. However, a vegetarian and, even more clearly, a vegan diet can lead to higher rates of fractures [11].

However, the impact of meat consumption on fracture risk in older individuals is unclear. Prevention of osteosarcopenia by a daily protein intake of 1.2–1.5 g protein/kg body weight for older adults has been recommended [10, 12]. In most Western populations, meat is the most common protein source [13], with an average daily intake of 50–100 g per person [14]. For health reasons, a maximum intake of 500 g of red and processed meat per week is recommended in current dietary guidelines [15, 16]. Meat is an important source of bioavailable nutrients, as well as protein. However, in addition to the potential to affect health, it influences the environment, which is reflected in the guideline on red and processed meat consumption in the Nordic Nutrition Recommendations 2023 [17, 18]. Meat, mainly processed red meat, has been implicated in inflammation, oxidative stress, and compromised immune function [14, 19].

We aimed to assess the association between meat intake and the risk of hip fractures in a prospective study of men and women, using data from two large cohorts with repeated data collection. We further explore biological mechanisms between meat intake and hip fracture risk by investigating associations between meat intake and clinical biomarkers of inflammation, bone turnover, and measures of BMD and body composition in two clinical sub-cohorts based on participants in the prospective cohorts.

## Methods

### Study cohorts

**Fig. 1:**
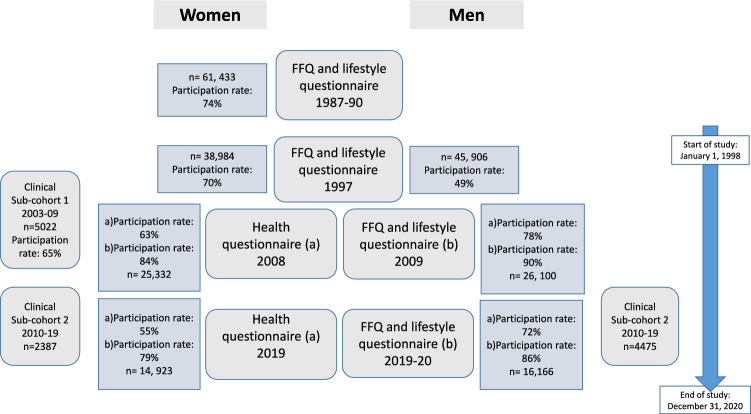
The flowchart depicts the number of participants and the participation rate in the different investigations in the Swedish mammography Cohort (women), the Cohort of Swedish men (men), and their respective sub-cohort

The present study population comprised of participants from two population-based cohort studies: The Swedish Mammography Cohort (SMC) and the Cohort of Swedish Men (COSM) and their respective sub-cohorts. The cohorts are part of the research infrastructure SIMPLER (http://www.simpler4health.se/). A flowchart with the number of participants and participation rate in each of the investigations is shown in Fig. 1.

The participating women and men live in three adjacent counties in central Sweden. SMC was established in 1987–1990 and COSM in late 1997. All women born between 1914 and 1948, residing in two counties (Uppsala and Västmanland), who were invited to a mammography screening (n = 90,303) also received an invitation to complete a questionnaire covering diet and lifestyle (n = 61,433). In the fall of 1997, a second, extended questionnaire was sent to all SMC participants (n = 56,030) still residing in the study area. All male residents (n = 100,303) of two counties (Örebro and Västmanland) born between 1918 and 1952 were invited to participate in COSM by completing a questionnaire similar to that for SMC. The 1997 questionnaire included almost 350 items on diet (food frequency questionnaire, FFQ) and other lifestyle factors (e.g., socio-demographic data, weight, height, total physical activity, self-perceived health status, smoking status, alcohol consumption, and use of dietary supplements). The investigations were repeated in 2008 and 2019 in both cohorts (Fig. 1). Both times a two-phase investigational approach was used; first, an invitation to complete the health questionnaire was sent out ((a) in Fig. 1), then only those who completed this questionnaire were invited to complete the FFQ and lifestyle questionnaire ((b) in Fig. 1). More information about the cohorts has been described by Harris et al. [20]. Participants were excluded from the analytical sample if the national registration number was missing, the questionnaire had not been dated, or energy intakes were deemed implausible (± 3 SD from the mean value of the log-transformed energy intake) at each follow-up. After these exclusions, the final analytical sample comprised 83,603 participants.

We also used data from the two clinical sub-cohorts formed by participants in the SMC and COSM. The sub-cohort participants underwent health examinations and completed additional questionnaires. The first sub-cohort (sub-cohort 1) included women from Uppsala taking part in SMC, and the second comprised participants from the neighbouring county of Västmanland; men participating in COSM and their spouses belonging to SMC (sub-cohort 2) (Fig. 1). In sub-cohort 1, the women were recruited between 2003 and 2009 as a random sample of SMC participants under the age of 85 years (born 1920–1948) living in Uppsala. Participants completed the questionnaire and took part in the health examination that included weight, height, waist, hip, blood pressure measurements, various blood samples, and a dual-energy X-ray absorptiometry (DXA, Lunar Prodigy, Lunar corp., Madison, WI, USA) scan (n = 5022). The health examination occurred 1–3 months after the questionnaire was completed. In sub-cohort 2, data were collected between 2010 through 2019 from participants of the COSM cohort, born 1920–1952 and living in Västerås. The investigation included a telephone-based cognitive test, completion of a questionnaire, and a health examination similar to that for the females, except for the DXA scan. Spouses from the SMC cohort were invited simultaneously to participate in the study. Althogether, 4475 COSM men and 2387 spouses, 85% of those invited, participated in sub-cohort 2. The regional ethics committees at Uppsala University, Uppsala, and Karolinska Institutet, Stockholm, Sweden, approved the investigations and our study (dnr 2018/261 och 2018/263). Informed consent was provided if the participant returned the questionnaires.

### Dietary assessment

The dietary assessment has been described previously [21]. The FFQs included 67, 96, 132, and 132 food items in 1987, 1997, 2009, and 2019, respectively. Participants indicated in the FFQs how often, on average, they had consumed each food item during the past year and chose from eight predefined frequency categories ranging from “never/seldom” to “3 or more times per day” (1997 FFQ). Frequently consumed foods (e.g., dairy products and bread) were also reported as daily servings. Further, information on the fat type used in cooking and salad dressing was reported. The total amount of alcohol consumed daily was derived from the FFQ by multiplying the reported frequency with the declared amount on a single occasion. Energy and nutrient intakes were estimated by multiplying the consumption frequency of each food item by the nutrient content of age-specific portion sizes. Nutrient values were obtained from the Swedish food composition database established by the Swedish Food Agency. The residual method adjusted nutrient intakes for total energy intake. Dietary data for the female sub-cohort was managed as described previously [22]. The reproduceability and validity of the estimated intake of nutrients, foods, and dietary patterns from the study FFQs have been assessed by comparison with multiple 24-h recall interviews, diet records and/or biomarkers [23–26]. For the intakes of processed meat, meat, and poultry compared with dietary records and in repeated FFQ, the correlations varied between 0.37 and 0.70 [23]. The correlations of total meat intake between the different investigations varied between 0.26 and 0.37.

### Examinations in the clinical sub-cohorts

#### Proteomics profiling and measurement of clinical biomarkers

Blood samples were drawn after overnight fasting, cool-centrifuged, light-protected, and frozen at − 80 °C until analysis. Analysis of plasma proteomics was performed using three high-throughput, multiplex immunoassays, the Olink Proseek^®^ Multiplex CVD 2, CVD 3, and Metabolism (Olink Bioscience, Uppsala, Sweden), as previously described [22]. The method has acceptable reproducibility and repeatability with a mean intra-assay coefficient of variation of approximately 8% and inter-assay variation of 12% [27]. Protein values below the limit of detection (LOD) were imputed as LOD/2 in sub-cohort 1 [22]; the machine output values were used in sub-cohort 2. Protein values were standardized to a distribution with a mean of 0 and SD of 1. The proteomics proteins used in the present analysis are 12 clinical biomarkers of inflammation and markers related to bone turnover. These include sclerostin (SOST), osteopontin (OPN), osteoprotegerin (OPG), insulin-like growth factor-binding proteins 1 and 2 (IGFBP-1 and -2), bone morphogenetic protein-6 (BMP-6), interleukin-6 (IL-6), fibroblast growth factor-21 (FGF-21), decorin (DCN), osteoclast-associated immunoglobulin-like receptor (hOSCAR), leptin (LEP), and growth/differentiation factor 15 (GDF-15).

In addition, total serum 25 hydroxyvitamin D, including 25-OHD_2_ and 25-OHD_3_, was assayed by high-performance liquid chromatography tandem mass spectrometry at Vitas, Oslo, Norway (www.vitas.no) [28]. Serum cross-laps and osteocalcin, as well as plasma C-reactive protein (CRP), parathyroid hormone (PTH), calcium, cystatin C, and alanine aminotransferase (ALAT), were analyzed using routine methods as described earlier [28]. Ferritin and transferrin were analyzed using standard methods. All clinical biomarkers were measured in sub-cohort 1, and CRP and calcium were measured in both.

#### Bone mineral density and body composition in sub-cohort 1

We measured BMD at the total dual hip (g/cm^2^), total body fat mass (FM, g), and lean mass (LM, g) by DXA (Lunar Prodigy, Lunar Corp, Madison, WI, USA) in women (n = 5022). The precision error of triple DXA scans on 15 individuals, including repositioning, was 0.8–1.5%, depending on the type of measurement (BMD, LM, or FM). The fat mass index (FMI) was calculated as the ratio of fat mass in kg divided by height in m squared (kg/m^2^). As previously described, osteoporosis was defined as a T-score at either the total hip, femoral neck, or spine ≤ 2.5 standard deviations (SD) below the mean value of young women [29].

### Exposure and covariates

The food groups used in the analyses (meat, fruits/vegetables, fish, chicken, milk, fermented milk, and cheese) were formed as summary variables of the food items belonging to the respective food groups using consumption frequency per day. Meat refers to total meat and reflects the consumption of red meat, processed meat, and chicken and other poultry. Meat intake was treated as servings per day and divided into quintiles (quintile models) and tertiles (in stratified analyses by intake of fruit and vegetables). The intake of fruits/vegetables was divided into tertiles. Tertiles of meat and fruits/vegetables were combined into nine joint strata. Information on relevant covariates was obtained from the questionnaires. BMI was calculated as weight (kg) divided by height squared (m^2^). Comorbidity, expressed as Charlson’s weighted comorbidity index [30, 31], was defined by International Classification of Diseases (ICD) codes (versions 8, 9, and 10) from the Swedish National Patient Register (NPR).

### Hip fractures

Our primary analysis considered outcomes between 1 January 1998 (study baseline) and 31 December 2020. In the SMC cohort, we also examined outcomes between the baseline in 1987–90 and 31 December 2020 in additional analyses. Hip fracture cases were defined by the ICD-10 codes (S720, S721, S722) and were obtained through individual linkage to the NPR [32]. The Swedish National Board of Health and Welfare has maintained the register that has covered all inpatient care in Sweden since 1987. Information from the registry enables complete follow-up of hip fractures [33–35].

### Statistical analysis

#### Cox regression models to investigate meat intake and risk for hip fracture

For each participant, we accrued follow-up time from baseline (1987–90 or 1 January 1998) until the first hip fracture, date of death, or the end of the study period (31 December 2020), whichever occurred first. The associations between quintiles of meat intake and per serving and hip fracture were investigated as age- and multivariable-adjusted hazard ratios (HRs) with 95% confidence intervals (CIs) using Cox proportional hazards regression models. Calendar date was used as the time scale. All variables, except educational level and age, were time-varying variables updated at the time of the 1997 (baseline for COSM), 2009, and 2019 investigations. Quintile 2 was used as the reference level for the quintile models to facilitate valid comparability with higher intake levels, as low and non-meat eaters may differ from meat eaters in several aspects of lifestyle behaviours [36]. In sensitivity analysis, the quintile models were also run with fixed data from 1997. To study potential nonlinear continuous associations, restricted cubic splines were used with three knots placed at the continuous exposure’s 10th, 50th, and 90th percentile [37]. The reference level for these models was set to the meat consumption frequency of once per day, roughly equal to the population median intake and the upper intake range of quintile 2.

The multivariable models included covariates that were chosen based on previous knowledge and directed acyclic graphs and included age, height (continuous), BMI (continuous), smoking habits (current, former, never), living alone (binary), educational level (≤ 9, 10–12, > 12 years, other), use of calcium and vitamin D supplements (binary), cortisone use (binary), walking/cycling (never/seldom, < 20 min/d, 20–40 min/d, 40–60 min/d, 1–1.5 h/d, > 1.5 h/d), leisure time physical exercise during the past year (< 1 h/w, 1 h/w, 2–3 h/w, 4–5 h/w, > 5 h/w), Charlson’s weighted comorbidity index, energy intake, intake of fruits/vegetables, and alcohol (all continuous). We re-ran these analyses to test for the multiplicative interaction to test the effect modification by fish intake (lower or higher than 1.75 times per week) or taking a supplement with vitamin D and/or calcium. In sensitivity analysis, the multivariable models included dairy products (milk, fermented milk, and cheese) or chicken or other poultry (continuous) as separate confounders. Thus, the model adjusting for chicken or other poultry reflects red and processed meat consumption. We further performed a sensitivity analysis, adding milk intake, fermented milk, cheese, chicken/poultry, and fish to the adjusted multivariable model. Using the cross-classified variable of meat and fruit/vegetable intake creating nine strata, a multivariable Cox proportional hazard analysis with a joint reference category (low meat/high fruits/vegetables) was run. We further tested whether there was an interaction effect of sex on the association between meat intake and hip fracture.

#### Linear regression models in the clinical sub-cohorts

##### Meat intake and protein and clinical biomarkers

Multivariable linear regression analyses were performed to examine associations between meat intake and biomarkers of inflammation, oxidative stress, and bone turnover in both sub-cohorts. The covariates in the multivariable model were age, physical activity level, metabolic equivalents of tasks (METS), educational attainment, energy intake, living alone, and cystatin C. The associations between meat intake and cross-laps (a reliable marker of bone resorption) and glomerular filtration rate (a marker of kidney function) were tested in a sensitivity analysis.

##### Meat intake, bone mineral density, and body composition

In sub-cohort 1, multivariable linear regression analysis was performed to identify associations between meat intake and BMD, and with LM, FM, and FMI, adjusted for age. The association with BMD was further adjusted for LM, FM, and height. We further tested for differences in meat intake according to the presence/absence of osteoporosis.

Missing data were multiple imputed using 20 imputations, taking into account model variables [38]. Most data of the main cohorts were missing for less than 1% of the participants for most variables. The variable with the most missing data was exercise (11%), followed by physical activity (9%), chicken intake (9%), living alone (6.5%), and BMI (4%). All analyses were performed with Stata, version 15.1 (StataCorp, College Station, TX, USA). Analyses were performed on resources provided by the Swedish National Infrastructure for Computing’s (https://www.snic.se/) support for sensitive data (SNIC-SENS) through the Uppsala Multidisciplinary Center for Advanced Computational Science (UPPMAX) under Project SIMP2021014.

## Results

### Background characteristics

The baseline characteristics of the 83,603 participants (54% men) in the 1997 investigation are listed in Table 1. The lowest quintile of meat intake corresponded to an average daily intake of 0.5 servings (~ 45 g) and the highest to 2.5 servings (~ 165 g). The 1997 investigation was the second for the women and the first for the men. The mean age of the participants was approximately 62 years. There were only minor differences across the quintiles of meat intake for most characteristics. However, the number of participants living independently was the highest in the lowest quintile of meat intake. Reported intakes of retinol, alcohol, protein, saturated fat, energy intake, and fruits and vegetables were higher in higher quintiles. For phosphorous and calcium, intake decreased in higher quintiles, but calcium averaged > 1000 mg/day within each quintile. The intake of chicken was low, thus most of the meat intake is red and processed meat.

**Table 1:** Background characteristics, stratified on quintiles of meat intake, of participants in the entire study at study start

	Quintile 1	Quintile 2	Quintile 3	Quintile 4	Quintile 5
Mean intake of meat (frequency per day (SD))	0.45 (0.18)	0.85 (0.12)	1.14 (0.14)	1.49 (0.17)	2.47 (1.13)
Gram per day (SD)	44 (21)	76 (24)	95 (28)	111 (34)	165 (117)
Number of participants	16, 125	16, 389	17, 743	16, 325	17, 021
Sex					
Female	7357 (45.6)	7059 (43.1)	8310 (46.8)	7786 (47.7)	7664 (45.0)
Male	8768 (54.4)	9330 (56.9)	9433 (53.2)	8539 (52.3)	9357 (55.0)
Age, mean years (SD)	63.6 (9.7)	61.4 (9.6)	60.5 (9.4)	60.9 (9.4)	61.5 (9.5)
Educational attainment					
≤ 9 years	6631 (41.3)	6151 (37.6)	6497 (36.7)	6122 (37.6)	6329 (37.3)
10–12 years	6693 (41.7)	7262 (44.4)	8021 (45.3)	7285 (44.7)	7804 (45.9)
> 12 years	2740 (17.1)	2942 (18.0)	3198 (18.1)	2887 (17.7)	2852 (16.8)
Height, mean cm (SD)	171 (8.8)	172 (8.8)	171 (8.8)	171 (8.8)	172 (9.0)
BMI, mean kg/m^2^ (SD)	25.3 (3.8)	25.3 (3.6)	25.5 (3.7)	25.5 (3.7)	25.7 (4.0)
Any calcium supplement*					
No	12,998 (80.6)	13,294 (81.1)	14,387 (81.1)	13,066 (80.0)	13,659 (80.2)
Yes	3127 (19.4)	3095 (18.9)	3356 (18.9)	3259 (20.0)	3362 (19.8)
Calcium supplement					
No	15,355 (95.2)	15,758 (96.1)	17,070 (96.2)	15,674 (96.0)	16,309 (95.8)
Yes	770 (4.8)	631 (3.9)	673 (3.8)	651 (4.0)	712 (4.2)
Any vitamin D supplement*					
No	13, 009 (80.7)	13, 306 (81.2)	14, 350 (80.9)	13, 048 (79.9)	13, 646 (80.2)
Yes	3116 (19.3)	3083 (18.8)	3393 (19.1)	3277 (20.1)	3375 (19.8)
Cortisone					
No	14, 518 (90.0)	14, 848 (90.6)	16, 012 (90.2)	14, 674 (89.9)	15, 187 (89.2)
Yes	1607 (10.0)	1541 (9.4)	1731 (9.8)	1651 (10.1)	1834 (10.8)
Exercise					
< 1 h/week	2793 (19.9)	3044 (20.8)	3392 (21.3)	3084 (20.9)	3367 (22.0)
1 h/week	2761 (19.7)	3122 (21.3)	3527 (22.1)	3284 (22.2)	3073 (20.1)
2–3 h/week	4536 (32.4)	4724 (32.2)	5234 (32.8)	4800 (32.5)	4860 (31.7)
4–5 h/week	1834 (13.1)	1841 (12.6)	1904 (11.9)	1749 (11.8)	1893 (12.4)
> 5 h/week	2095 (14.9)	1926 (13.1)	1903 (11.9)	1845 (12.5)	2128 (13.9)
Walking					
Never/seldom	1857 (12.9)	1813 (12.1)	1980 (12.1)	1825 (12.2)	2045 (13.0)
< 20 min/day	2962 (20.6)	3277 (21.9)	3685 (22.6)	3328 (22.2)	3392 (21.6)
20–40 min/day	4345 (30.2)	4779 (32.0)	5300 (32.5)	4775 (31.8)	4857 (31.0)
40–60 min/day	2446 (17.0)	2512 (16.8)	2708 (16.6)	2610 (17.4)	2533 (16.1)
1–1.5 h/day	1519 (10.6)	1427 (9.5)	1460 (9.0)	1335 (8.9)	1542 (9.8)
> 1.5 h/day	1268 (8.8)	1142 (7.6)	1172 (7.2)	1122 (7.5)	1324 (8.4)
Smoking status					
Current	3924 (24.9)	3763 (23.3)	4157 (23.7)	3941 (24.5)	4056 (24.2)
Former	4807 (30.5)	5257 (32.6)	5640 (32.2)	5043 (31.3)	5316 (31.7)
Never	7029 (44.6)	7115 (44.1)	7713 (44.0)	7129 (44.2)	7409 (44.2)
Charlson’s comorbidity index					
0	12, 926 (80.2)	13, 810 (84.3)	14, 940 (84.2)	13, 633 (83.5)	13, 880 (81.5)
1	1934 (12.0)	1552 (9.5)	1662 (9.4)	1643 (10.1)	1900 (11.2)
≥ 2	1265 (7.8)	1027 (6.3)	1141 (6.4)	1049 (6.4)	1241 (7.3)
Living alone					
No	10, 895 (72.4)	12, 248 (79.5)	13, 599 (82.0)	12, 554 (82.3)	13, 029 (81.7)
Yes	4158 (27.6)	3160 (20.5)	2991 (18.0)	2707 (17.7)	2912 (18.3)
Mean daily intakes of residual-adjusted nutrients (SD)					
Vitamin D (µg)	5.5 (3.3)	5.6 (2.6)	5.6 (2.3)	5.7 (2.4)	6.0 (2.6)
Retinol (g)	0.86 (0.5)	0.97 (0.6)	1.0 (0.6)	1.1 (0.7)	1.4 (1.3)
Phosphorous (mg)	1826 (484)	1805 (449)	1752 (435)	1726 (428)	1714 (417)
Protein (g)	85.9 (21.6)	88.3 (20.6)	87.6 (20.3)	87.5 (20.3)	90 (21.2)
Alcohol (g)	6.0 (8.7)	7.2 (8.4)	7.6 (8.7)	7.9 (9.0)	8.7(11)
Saturated fat (g)	30 (16)	33 (16)	35(16)	37 (17)	43 (20)
Calcium (mg)	1404 (526)	1335 (455)	1263 (427)	1216 (408)	1145 (388)
Energy (kcal)	1878 (759)	2124 (772)	2212 (786)	2317 (810)	2656 (1050)
Mean daily consumption of food groups (frequency per day) and SD					
Meat	0.45 (0.18)	0.85 (0.12)	1.14 (0.14)	1.49 (0.17)	2.47 (1.13)
Chicken	0.07 (0.05)	0.08 (0.06)	0.09 (0.07)	0.10 (.08)	0.14 (0.2)
Cheese	2.8 (2.3)	2.9 (2.3)	2.9 (2.2)	2.9 (2.2)	2.9 (2.2)
Fermented milk	0.8 (1.0)	0.8 (1.0)	0.8 (0.97)	0.8 (1.0)	0.8 (1.02)
Milk	1.2 (1.4)	1.3 (1.4)	1.3 (1.4)	1.3 (1.4)	1.4(1.4)
Fish and shellfish	0.4 (0.4)	0.5 (0.4)	0.5 (0.4)	0.5 (0.4)	0.7 (0.7)
Fruits and vegetables	3.9 (2.9)	4.1 (2.4)	4.3 (2.3)	4.6 (2.5)	5.2 (3.1)

Number of participants (percentage) is shown if not otherwise indicated

*BMI* Body mass index, *SD* standard deviation

*Including supplements with only the nutrient or as taken in multi-vitamin-mineral supplements

### Time to hip fracture

**Fig. 2:**
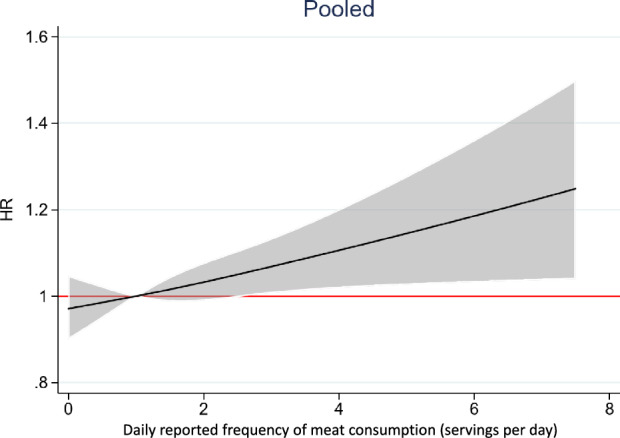
Associations between meat intake (mainly red and processed meat, since chicken and other poultry only account for a small portion of meat intake) and hip fracture risk in the pooled sample of women and men. Depicted are the multivariable-adjusted hazard ratios and 95% confidence intervals (shaded) of hip fractures. The variable meat was time-updated in 2009 and 2019 and modelled as a continuous exposure using a restricted cubic spline Cox model. The multivariable analysis included age, height, Body Mass Index, smoking habits, living alone, educational level, use of calcium and vitamin D supplement, cortisone use, walking/cycling, leisure time physical exercise during the past year, Charlson’s weighted comorbidity index, energy intake, intake of fruits/vegetables and alcohol.. HR hazard ratio

In our main analysis, 7345 participants (4505 women) experienced a hip fracture during up to 23 years of follow-up (mean 18.2 years) and 1,538,627 person-years at risk. The risk of hip fracture increased with increasing meat intake and the analyses indicated a linear association. The age- and multivariable-adjusted HRs with 95% CIs are shown in Table 2. The multivariable-adjusted HR was 1.03 per quintile step (95% CI 1.00; 1.06; p = 0.02) and 1.11 (95% CI 1.01; 1.21) in quintile 5 compared to quintile 2. We found no interaction with sex (*p* = 0.66) on the association. The multivariable-adjusted Cox model using restricted cubic splines and time-updated variables confirmed the linear relationship (Fig. 2) in the overall pooled sample. The results were similar in men and women (Supplemental Fig. 1) but with more pronounced estimates in women. In women, we performed an additional analysis using data from 10 years before the baseline used in the main analysis, thus including data from all four investigations (1987, 1997, 2009, 2019). Results from this analysis, shown in Supplemental Fig. 2, revealed a similar pattern to the main analysis shown in Supplemental Fig. 1. This analysis had a longer follow-up and included 7027 women with an incident hip fracture, 64% more hip fractures than with baseline in 1997. The multivariable-adjusted HR per serving (around 80 g) was 1.04 (95% CI 1.01; 1.07). In quintile 5, compared to quintile 2, the multivariable HR of hip fracture was 1.11 (95% CI 1.0; 1.22).

**Table 2:** The table shows the multivariable-adjusted hazard ratio (HR) and 95% confidence intervals (CI) of hip fracture in quintiles of time-updated meat intake

	Quintile 1	Quintile 2	Quintile 3	Quintile 4	Quintile 5	Per serving
Mean intake of meat (frequency per day (SD))	0.45 (0.18)	0.85 (0.12)	1.14 (0.14)	1.49 (0.17)	2.47 (1.13)	
Overall						
Number of persons with hip fracture	1228	1002	967	957	1122	
Person-years of follow-up	255,420	265,547	282,598	266,193	268,237	
Crude rate/1000 person-years (95% CI)	4.8 (4.5; 5.1)	3.8 (3.5; 4.0)	3.4 (3.2; 3.6)	3.6 (3.4; 3.8)	4.1 (3.9; 4.4)	
Age-adjusted HR (95% CI)	1.05 (0.97, 1.14) *P* = 0.23	1.00 (ref)	1.00 (0.91, 1.08)	1.01 (0.92, 1.10)	1.08 (0.98, 1.18)	1.02 (1.00; 1.05) *P* = 0.08
			*P* = 0.25	*P* = 0.81	*P* = 0.09	
Multivariable-adjusted HR (95% CI)*	1.00 (0.92, 1.08) *P* = 0.99	1.00 (ref)	1.02 (0.94, 1.11)* P* = 0.64	1.05 (0.97, 1.15) *P* = 0.25	1.11 (1.01, 1.21) *P* = 0.03	1.03 (1.00; 1.06) *P* = 0.02
Women						
Number of persons with hip fracture	719	593	587	601	687	
Person-years of follow-up	116,789	117,588	132,518	125,873	121,958	
Crude rate/1000 person-years (95% CI)	6.2 (5.7; 6.6)	5.0 (4.7; 5.5)	4.4 (4.1; 4.8)	4.8 (4.4; 5.2)	5.6 (5.2; 6.1)	
Age-adjusted HR (95% CI)	1.04 (0.94, 1.16) *P* = 0.41	1.00 (ref)	1.00 (0.90, 1.12)	1.04 (0.93, 1.16)	1.10 (0.99, 1.23)	1.03(1.00, 1.06) *P* = 0.09
			*P* = 0.90	*P* = 0.48	*P* = 0.07	
Multivariable-adjusted HR (95% CI)*	1.00 (0.90, 1.11) *P* = 0.97	1.00 (ref)	1.03 (0.92, 1.15)* P* = 0.64	1.08 (0.97, 1.21)* P* = 0.18	1.13 (1.01, 1.26)* P* = 0.03	1.04 (1.00, 1.08) *P* = 0.03
Men						
Number of persons with hip fracture	509	409	380	356	435	
Person-years of follow-up	138,631	147,959	150,080	140,32	146,279	
Crude rate/1000 person-years (95% CI)	3.6 (3.4; 4.0)	2.8 (2.5; 3.0)	2.5 (2.3; 2.8)	2.5 (2.3; 2.8))	2.9 (2.7; 3.3)	
Age-adjusted HR (95% CI)	1.06 (0.93, 1.20) *P* = 0.38	1.00 (ref)	0.98 (0.85, 1.12)	0.96 (0.84, 1.10)	1.04 (0.91, 1.19)	1.02 (0.98, 1.06) *P* = 0.37
			*P* = 0.71	*P* = 0.59	*P* = 0.57	
Multivariable-adjusted HR (95% CI)*	0.99 (0.87, 1.13) *P* = 0.88	1.00 (ref)	1.00 (0.88, 1.15) *P* = 0.94	1.00 (0.87, 1.15)* P* = 0.98	1.04 (0.90, 1.20)* P* = 0.56	1.02 (0.98, 1.06) *P* = 0.37

The results are shown for the overall sample, as well as in women and men

*HR* Hazard ratio, *CI* confidence interval

**Fig. 3:**
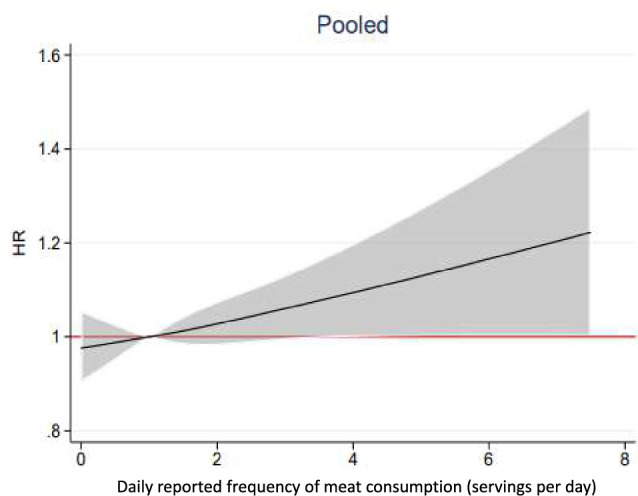
Associations between meat intake (mainly red and processed meat, since chicken and other poultry only account for a small portion of meat intake) and hip fracture risk in the pooled sample of women and men. Depicted are the multivariable-adjusted hazard ratios and 95% confidence intervals (shaded) of hip fractures. The variable meat was time-updated in 2009 and 2019 and modelled as a continuous exposure using a restricted cubic spline Cox model. The multivariable adjusted analysis included age, height, Body Mass Index, smoking habits, living alone, educational level, use of calcium and vitamin D supplement, cortisone use, walking/cycling, leisure time physical exercise during the past year, Charlson’s weighted comorbidity index, energy intake, intake of fruits/vegetables, alcohol, milk, fermented milk, cheese, chicken/poultry and fish. HR hazard ratio

In additional analyses, we did not find indications of effect modification either by fish intake (interaction *p*-value 0.66), vitamin D, or calcium supplement use (interaction *p*-value 0.28) (data not shown). In further sensitivity analysis, we adjusted for the intake of dairy products (Supplemental Fig. 3) and chicken or other poultry (Supplemental Fig. 4), which did not change the association between meat intake and hip fracture. The fully adjusted multivariable adjusted model, additionally including milk, fermented milk, cheese, chicken/poultry and fish (Fig. 3) gave similar results as the main analysis (Fig. 2). The sensitivity analysis using only data from the 1997 investigation, without later time-updated information, confirmed the results (data not shown).

**Fig. 4:**
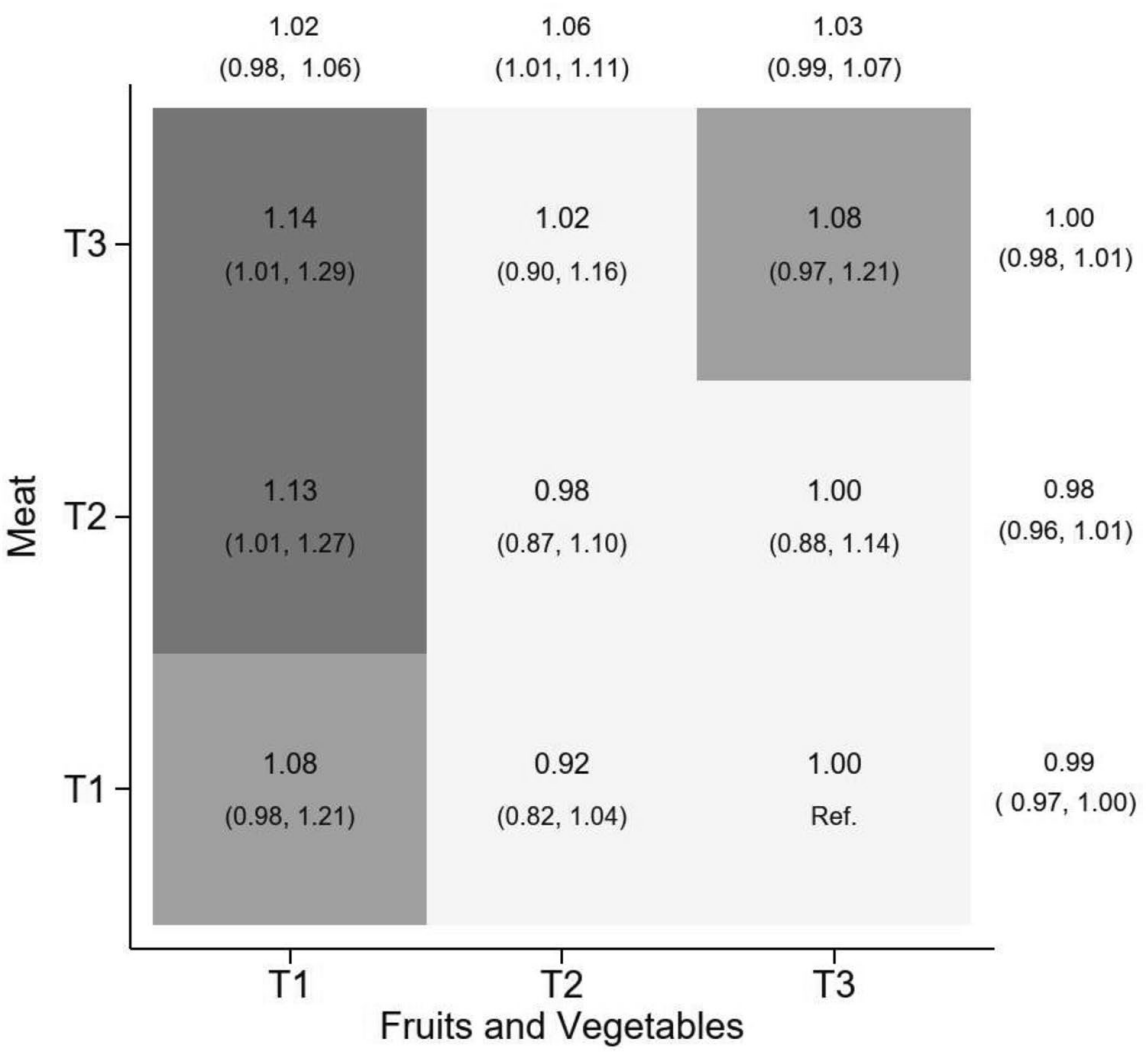
Depicted in the heat map are the multivariable-adjusted hazard ratios and 95% confidence intervals for the association between the combined tertiles of frequencies of the intake of meat and fruits and vegetables across nine cross-classified strata and hip fracture in the pooled sample. The multivariable analysis included the covariates described in the Statistical analysis. P_i._ P-value for interaction; P_c_, The combined P-value

The results from the joint analysis displayed in the heat map in Fig. 4 illustrate the associations for hip fracture between the combined categories of meat intake and fruits and vegetables across nine strata, with the low meat/high fruits and vegetables category as the reference group. The graph also displays trends for HRs of hip fracture within tertiles of meat and fruit/vegetable intake. The results revealed no interaction between the two food groups on hip fracture risk (*p*-value for interaction 0.90).

### Meat intake, circulating proteins, and clinical biomarkers

Associations between meat intake and circulating protein and clinical biomarkers investigated in multivariable-adjusted linear regression analyses are presented in Table 3. Meat intake was directly associated with serum IL-6, FGF-21, and leptin (LEP) and inversely with IGFBP-1 and IGFBP-2 in both sexes. Meat intake was also directly associated with OPG and GDF-15, but only in men. Further, meat intake was directly associated with ferritin, CRP, Cystatin-C, PTH, calcium levels, and ALAT and inversely associated with osteocalcin. The sensitivity analysis found no association between meat intake and s-cross laps or GFR (data not shown).

**Table 3:** Associations between meat intake and clinical biomarkers of inflammation, oxidative stress, and bone turnover

		Sub-cohort 1 Women, n = 4656								Sub-cohort 2 Men and women, n = 6862									Sub-cohort 2 Men, n = 4475				
		Crude				Adjusted				Crude					Adjusted				Crude			Adjusted	
		*β*-coeff		*p*		*β*-coeff		*p*		*β*-coeff			*p*		*β*-coeff		*p*		*β*-coeff		*p*	*β*-coeff	*p*
BMP-6	Bone morphogenetic protein 6	− 0.01		0.27		− 0.06		0.49		0.04			0.009		0.018		0.22		0.04		0.05	0.013	0.49
IL-6	Interleukin-6	0.025		0.007		0.032		0.001		0.06			< 0.001		0.06		< 0.001		0.05		0.01	0.05	0.03
FGF-21	Fibroblast growth factor 21	0.024		0.009		0.035		< 0.001		0.06			< 0.001		0.08		< 0.001		0.06		< 0.001	0.08	< 0.001
DCN	Decorin	− 0.009		0.33		− 0.008		0.40		0.04			0.003		0.02		0.18		0.05		0.002	0.03	0.06
hOSCAR	Osteoclast-associated immunoglobulin-like receptor	− 0.006		0.54		− 0.015		0.13		0.03			0.04		0.014		0.37		0.05		0.009	0.03	0.11
LEP	Leptin	0.044		< 0.001		0.053		< 0.001		0.06			< 0.001		0.10		0.04		0.09		< 0.001	0.12	< 0.001
OPG	Osteoprotegerin	− 0.009		0.03		− 0.017		0.08		0.03			0.02		0.007		0.56		0.03		0.02	0.007	0.04
GDF-15	Growth/differentiation factor 15	0.006		0.59		0.002		0.83		0.09			< 0.001		0.09		< 0.001		0.078		< 0.001	0.082	< 0.001
OPN	Osteopontin	− 0.002		0.83		− 0.01		0.26		0.04			0.001		0.03		0.05		0.04		0.008	0.02	0.23
IGFBP-1	Insulin-like growth factor-binding protein 1	− 0.045		< 0.001		− 0.052		0.001		− 0.02			0.08		− 0.04		0.002		− 0.03		0.04	-0.07	< 0.001
IGFBP-2	Insulin-like growth factor-binding protein 2	− 0.046		< 0.001		− 0.054		0.002		− 0.03			0.01		− 0.04		0.002		− 0.03		0.01	-0.04	0.002
SOST	Sclerostin	− 0.004		0.63		− 0.004		0.62		0.04			0.005		0.02		0.1		0.03		0.05	0.02	0.22
	Total 25 (OH) vitamin-D	− 0.007		0.14		− 0.003		0.5															
	Ferritin	0.09		< 0.001		0.1		< 0.001															
	Transferrin	− 0.08		0.21		− 0.09		0.15															
CRP	C-Reactive Protein	0.039		< 0.001		0.045		< 0.001		0.06			< 0.001		0.06		< 0.001		0.05		< 0.001	0.05	< 0.001
PTH	Parathyroid hormone	0.07		0.010		0.06		0.02															
	Plasma calcium	0.33		0.09		0.4		0.04		0.34			0.009		0.43		0.001		0.37		0.03	0.04	0.01
	Osteocalcin	− 0.002		0.06		− 0.002		0.03															
ALAT	Alanine aminotransferase	0.038		0.05		0.044		0.02															

The table shows the results from the crude and multivariable-adjusted linear regression analyses in the clinical sub-cohorts 1 and 2

Adjusted for Cystatin C, physical activity level, educational level, age, and energy intake. Living alone was also included in the analysis, but only for women. Missing data of covariates were imputed using multiple imputation

### Meat intake, body composition, and bone mineral density

The multivariable linear regression analysis with age as the only covariate showed positive associations between meat intake and FM, LM, and FMI. The analysis also showed an association between meat and bone mineral T-score (P < 0.001 for all). The association with T-score was lost after FM, LM, and height adjustment. Furthermore, meat intake in those classified as osteoporotic (0.98 servings per day) or not (1 serving per day) was numerically similar but statistically different (P = 0.004).

## Discussion

This large prospective study of women and men revealed a modest positive linear association between meat intake—primarily red and processed meat—and hip fracture rate. This association was found in both sexes, albeit with more robust estimates in women. The association was independent of several lifestyle factors, comorbidity, and other foods. We could not find an indication of effect modification by fish intake or calcium or vitamin D supplement use. A higher intake of fruits and vegetables did not counteract the higher risk of hip fracture with higher meat intake. However, increasing the consumption of fruit and vegetables with up to five servings daily is associated with reduced hip fracture risk dose-dependently [39, 40]. Although the HR estimates are modest, limiting red and processed meat intake in a healthy diet is advisable to prevent hip fractures. It also contributes to a shift towards a more environmentally sustainable diet.

While a Western dietary pattern higher in meat is related to a higher risk of hip fracture [41], no large cohort study has previously investigated the association between meat intake per se and hip fracture. A small case–control study from China found an odds ratio for the highest vs. lowest quartile of red meat of 2.94 (95% CI 1.82; 4.76) [42]. The researchers linked this effect to fatty pork and organ meat intake since the study showed no association between poultry intake and hip fracture.

Mechanisms that could explain our findings include the effect of protein on bone, markers of inflammation and oxidative stress, or other effects on bone turnover, as indicated in our analyses in the sub-cohorts. There has been an ongoing debate on whether protein is detrimental or beneficial for fracture risk prevention, and previous systematic reviews have presented divergent results. One review reported an inverse association between total protein intake (but not vegetable or animal protein separately) and hip fracture [43], while another review reported no association between protein intake and hip fracture risk [44]. Moreover, a review from 2017 concluded that a negative association between meat intake and bone health was observed in a Western diet but not in a Mediterranean or Asian diet [45]. A beneficial effect of protein could be explained by increases in circulating levels of insulin-like growth factor 1 [46] and detrimental effects of protein by reduced body pH, which increases bone resorption. Reduced body pH is associated with increased calciuria [47]. However, this acid-ash diet hypothesis of osteoporosis has not been verified [48]. Adjustments for other protein sources in the present study did not affect the results.

The risk of hip fracture is also influenced by the risk of falls, which is affected by the presence of sarcopenia [6, 7]. Older people are recommended to consume enough protein to prevent both osteoporosis and sarcopenia [10, 12]. A previous study from the UK concluded that dietary patterns with characteristics of a traditional British diet, including high intake of red meat, was associated with an increased risk of sarcopenia even when participants had an adequate protein intake [49]. This further supports the results of the present study, since it is known that oxidative stress and inflammation are implicated in both age-related bone loss and sarcopenia [6, 7]. A large cohort study (85 871 women aged ≥ 60 years) showed that a higher intake of red and processed meat increases the risk of frailty [50], which is also known to increase the risk of falls.

Previous studies that have compared differences in hip fracture rates in meat to non-meat eaters have reported an increased risk among non-meat eaters. In the Epic-Oxford cohort (55,000 participants, 945 hip fractures) vegans, compared to meat eaters, had higher risks of total, hip, leg, and vertebral fractures, and fish eaters and vegetarians had a higher risk of only hip fractures. Similar results were observed in the Adventist Health Study 2 (US and Canada, 34,542 participants, 679 hip fractures) [51] and the UK Women’s Cohort Study (26, 318 participants, 822 hip fractures) [52]. In other studies, people adhering to vegetarian diets have been found to have lower BMD [53, 54].

The higher risk of hip fracture observed in populations that exclude meat from the diet [11, 51] may be related to dietary factors other than meat. Higher adherence to healthy diets, such as Mediterranean diets, including a moderate meat intake, is associated with a lower risk of hip fracture [55, 56]. Vegetarian diets that exclude all or specific animal foods are lower in nutrients essential for bone health, such as protein, calcium, vitamin D, zinc, and vitamin B_12_. Nonetheless, a vegetarian diet may also be higher in certain nutrients necessary for bone health, such as potassium, magnesium, and vitamins C and K [57], but also higher in levels of hazardous substances (e.g., cadmium), which have been associated with an increased risk of fracture [58]. In our study, the estimates were adjusted for the intake of fruits and vegetables. We found that those with a higher meat intake also had a higher intake of fruits and vegetables and higher protein and saturated fat intake, while calcium intake decreased. However, the association of meat intake with the risk of hip fracture was independent of the consumption of dairy products [59].

### Meat consumption and markers of inflammation and oxidative stress

Inflammatory cytokines (e.g., tumor necrosis factor-alpha [TNF]-α and IL-1, IL-6, and IL-17) promote the generation of osteoclasts and their activity while simultaneously inhibiting osteoblast differentiation and function [60], which may lead to increased bone resorption. We found that meat consumption was positively associated with levels of IL-6 and CRP in men and women in the sub-cohorts. Elevated levels of the acute-phase protein CRP over an extended period have been linked to higher bone loss rates [61]. We show a direct association between meat intake and serum ferritin in women, consistent with evidence that meat significantly contributes to iron content in the Swedish diet [62]. In excess, iron can act as an oxidative stressor because free iron can catalyze the generation of highly reactive free radicals with the capacity to damage biomolecules and cells. Oxidative stress affects bone [63] by several mechanisms, including promoting bone cell apoptosis [60]. Serum ferritin has been suggested as a marker of iron-related oxidative stress [64] and was positively correlated with CRP and IL-6 in the current study (data not shown).

### Meat intake and bone turnover

Meat intake was negatively associated with insulin-like growth factor-binding proteins 1 and 2 (IGFBP) and osteocalcin levels in the sub-cohorts. IGFBPs modulate the action of insulin growth factors essential for bone, stimulate osteoblastic cell proliferation and protein synthesis, and are expressed by active osteoblasts [46]. Osteocalcin is also involved in bone formation: serum levels are higher at a higher bone loss (e.g., post-menopausal women) [65]. Thus, the observed inverse association between meat intake and levels of IGFBP 1 and 2 and osteocalcin is consistent with our finding of a linear association between meat and hip fracture risk.

Meat intake and levels of FGF-21 and leptin were associated positively in both sexes. FGF21 is a fibroblast growth factor superfamily member regulating energy metabolism, especially fat and carbohydrate metabolism [66]. Human studies suggest a negative association between circulating FGF21 and BMD via inhibition of osteoblast activity and enhanced osteoclast activity [67], but the evidence is limited. Leptin, a hormone involved in energy balance, also regulates bone mass. Increased leptin levels may lead to osteoblast signalling to stimulate bone resorption, thereby inhibiting bone formation [68].

The present analysis also indicated a positive association between meat intake and PTH in women, and meat intake and calcium levels in both sexes. Parathyroid hormone (PTH) maintains normal serum calcium levels, and when levels are low, PTH levels rise. A positive association between meat and levels of serum PTH and calcium is consistent with higher bone resorption [69]. There was an association between meat intake and OPG that was positive in men, whereas it was negative and borderline significant in women, complicating the interpretation of our findings.

Our results based on the analyses in the sub-cohorts provide evidence that a higher meat intake may contribute to an increased risk of hip fractures by inducing inflammation and oxidative stress or influencing the regulation of bone turnover while slightly affecting BMD. In women, meat intake was similar in those classified as osteoporotic compared to those not classified, and was not associated with BMD independently of measures of body composition. However, since these analyses were performed on cross-sectional data, we cannot draw any firm conclusions based on causality.

Our study has both strengths and weaknesses. This is the first large cohort study investigating meat intake and hip fracture risk. Another major strength is that the two population-based studies include a large number of hip fractures in both sexes, allowing precise assessment of hip fracture risks. It was also possible to achieve complete ascertainment of hip fractures using nationwide patient registers with no loss to follow-up. The study updated the diet exposure three times in women and twice in men, which is a major strength. The latest update was in 2019, ensuring up-to-date information on diet among participants that were censored at the end of the study (31 December 2020). Sensitivity analyses using fixed data confirmed the results as well. Although the multivariable analyses included many important covariates, residual or unmeasured confounding may occur. The collection of diet data is inherently prone to some limitations. However, even if the absolute amounts estimated by an FFQ are underestimated, the ranking of study participants is retained. It will not bias HR estimates when one level is compared with another. The large study size will compensate for random misclassification [70]. Although the investigations took place ten years apart, estimated intake of meat correlated between the different investigations with correlation coefficients 0.26 to 0.37. The clinical sub-cohorts also provide essential information, including several clinical biomarkers of inflammation and bone turnover, measured BMD, and body composition.

In conclusion, the present study indicates that a higher total meat intake is linearly associated with an increased risk of hip fractures in men and women. Our results from our analyses in the sub-cohorts further suggest underlying mechanisms, including that meat intake may influence the regulation of bone turnover, subclinical inflammation, and oxidative stress. Although estimates are modest, limiting meat intake in a healthy diet is advisable to prevent hip fractures. The present findings should be confirmed in other cohorts, and the underlying mechanism of the association should be determined.

### Supplementary Information

Below is the link to the electronic supplementary material.

Supplementary file1 (PDF 796 KB)

## Data Availability

More information on how to request data from SIMPLER, https://www.simpler4health.se. Ethical approval is required for access to SIMPLER data.

## Abbreviations

ALAT: Alanine aminotransferase
BMD: Bone mineral density
BMP-6: Bone morphogenetic protein-6
COSM: Cohort of Swedish men
CRP: C-reactive protein
DCN: Decorin
DXA: Dual-energy X-ray absorptiometry
FFQ: Food frequency questionnaire
FGF-21: Fibroblast growth factor-21
FM: Fat mass
FMI: Fat mass index
GDF-15: Growth/differentiation factor 15
hOSCAR: Osteoclast-associated immunoglobulin-like receptor
IGFBP-1 and -2: Insulin-like growth factor-binding proteins 1 and 2
IL-6: Interleukin-6
LEP: Leptin
LM: Lean mass
LOD: Limit of detection
METS: Metabolic equivalents of tasks
OPG: Osteoprotegerin
OPN: Osteopontin
PTH: Parathyroid hormone
SOST: Sclerostin
SMC: Swedish mammography cohort
