# Supplementary material for: Meat consumption and the risk of hip fracture in women and men: two prospective Swedish cohort studies
Source: Eur J Nutr. 2024 Apr 17;63(5):1819–33. doi: 10.1007/s00394-024-03385-z (PMC11329405; doi:10.1007/s00394-024-03385-z)
Supplement: Supplementary file 1 — Supplementary file1 (PDF 796 KB) [file 394_2024_3385_MOESM1_ESM.pdf]

Supplementary Information, supplemental figures 1-4

Meat consumption and the risk of hip fracture in women and men: Two prospective Swedish cohort studies

Eva Warensjö Lemming, Liisa Byberg, Jonas Höijer, John A Baron, Alicja Wolk and Karl Michaëlsson

Supplemental figure 1

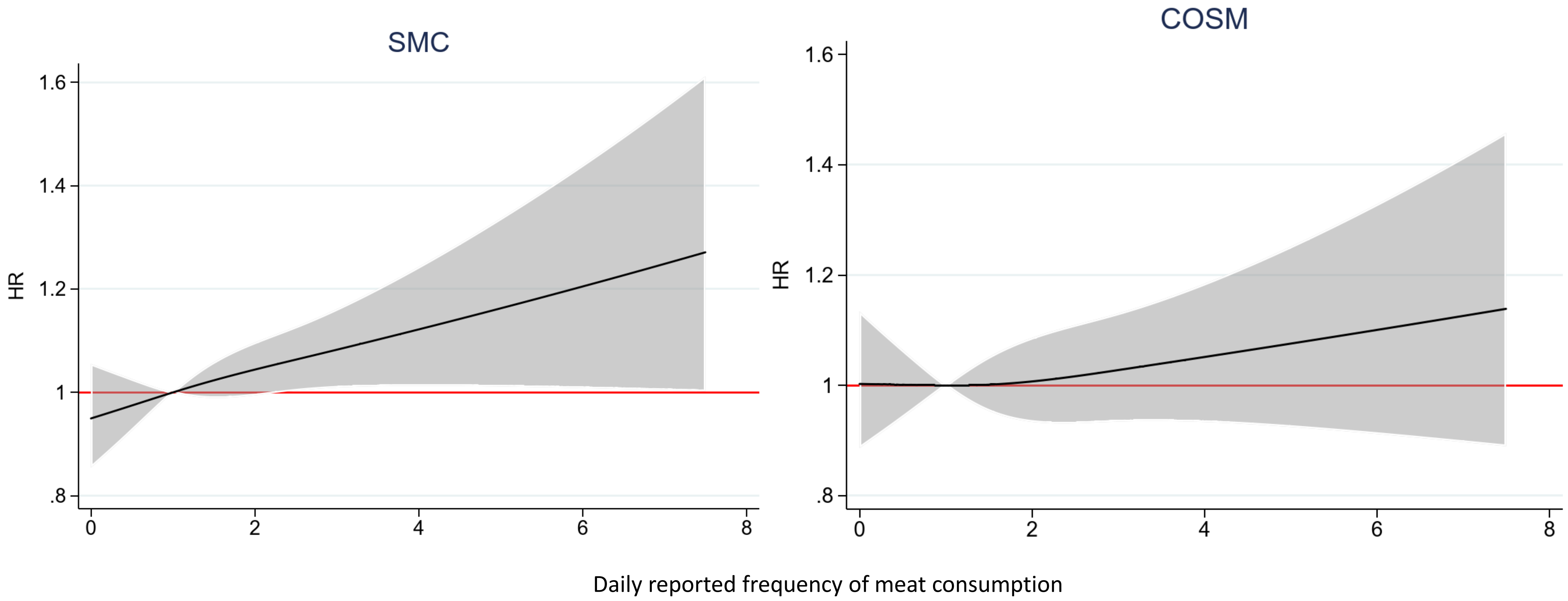

HR Hazard Ratio SMC Swedish mammography cohort COSM Cohort of Swedish men

Supplemental figure 2

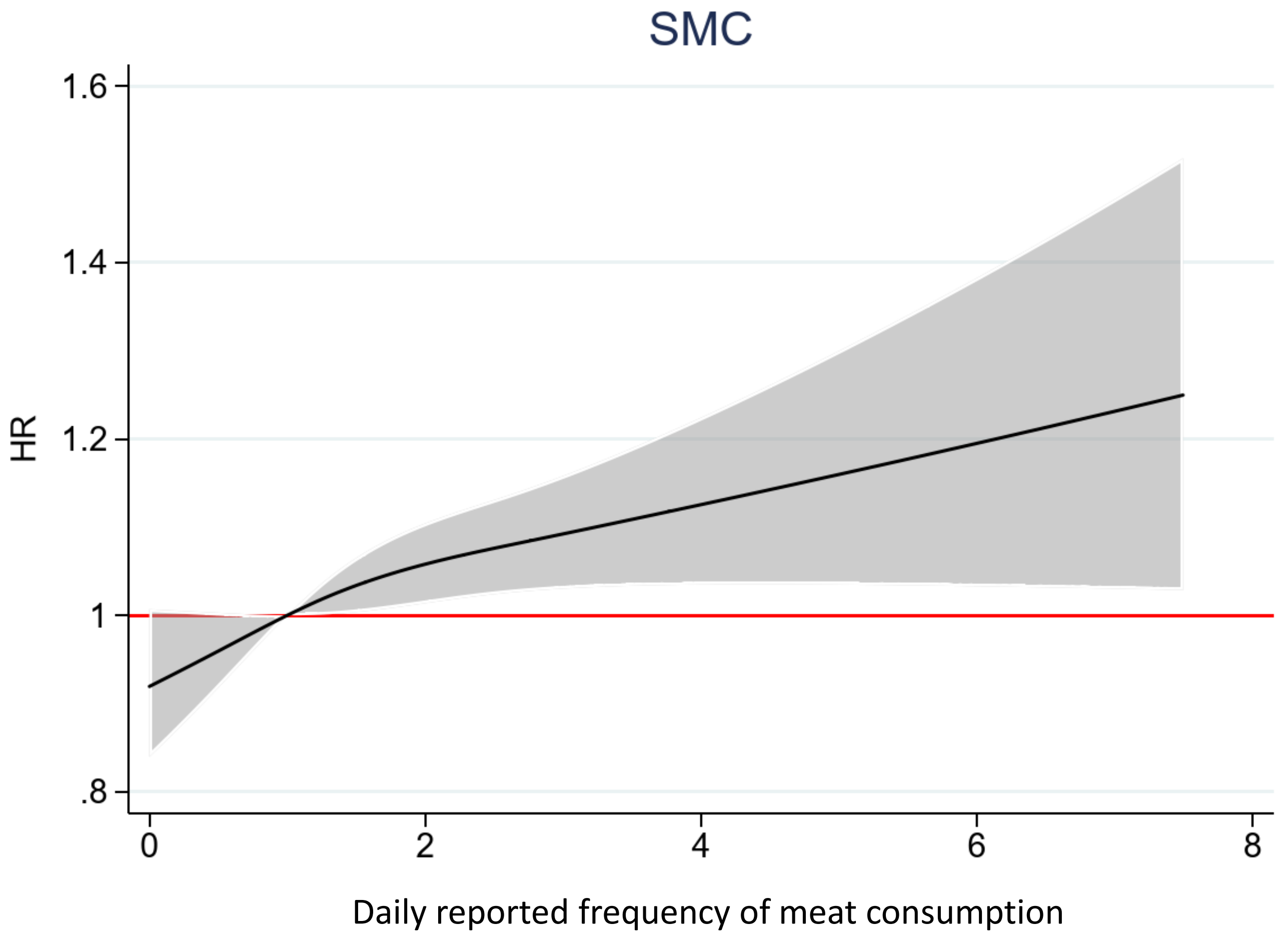

HR Hazard Ratio SMC Swedish mammography cohort

Supplemental figure 3

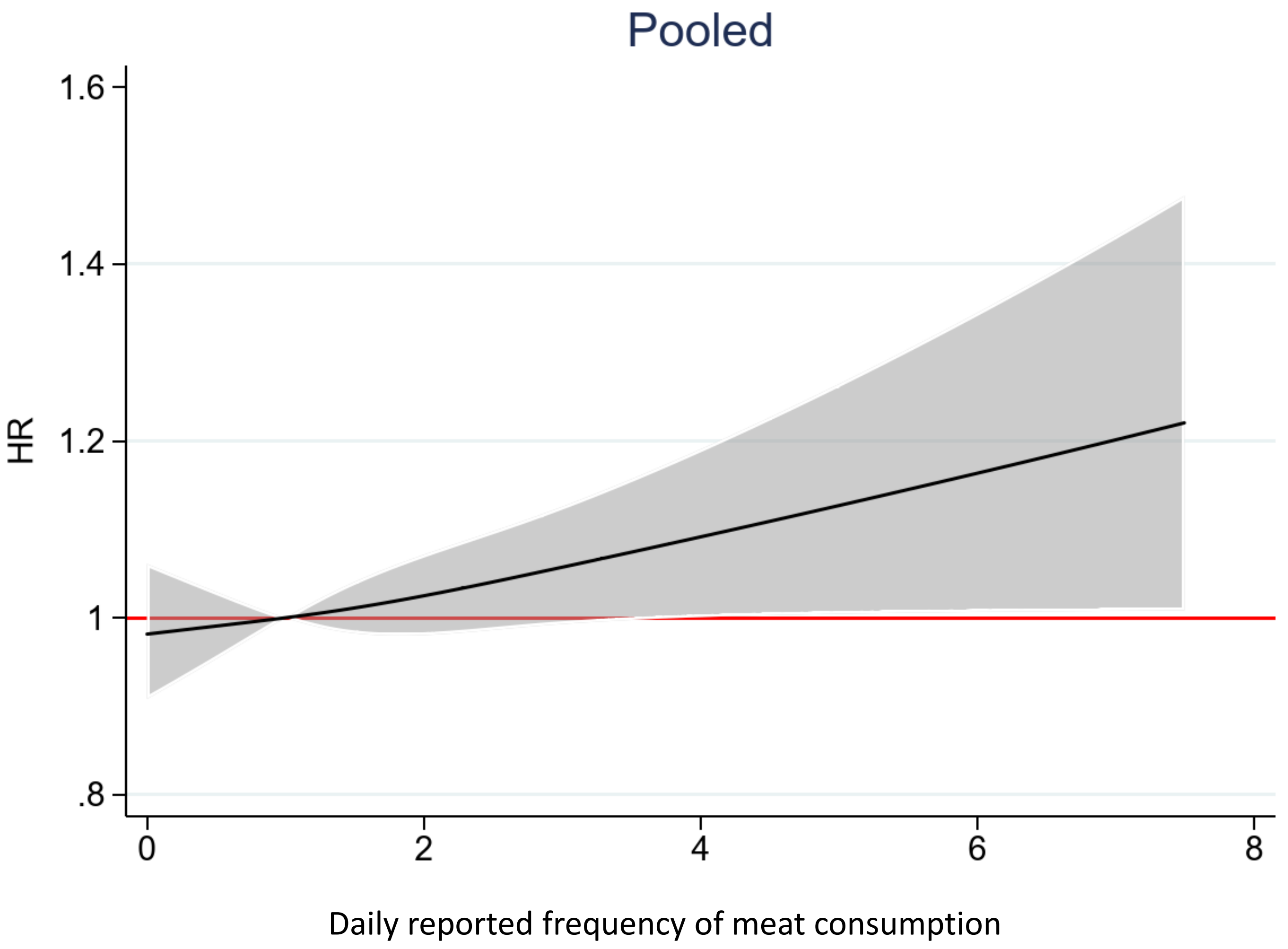

HR Hazard Ratio, pooled sample of both women and men

Supplemental figure 4

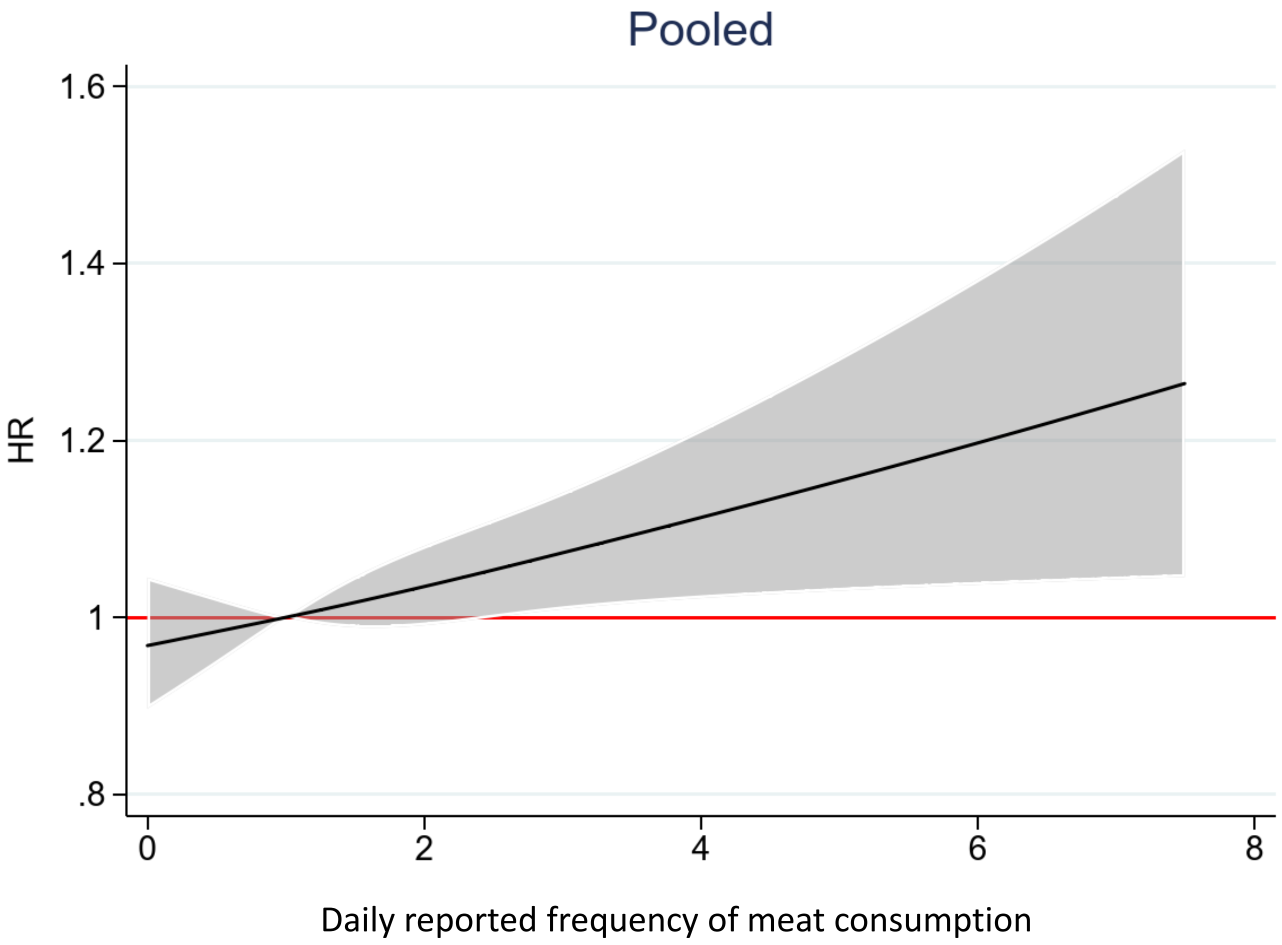

HR Hazard Ratio, pooled sample of both women and men

Figure legends

Supplemental figure 1. Associations between meat intake (mainly red and processed meat, since chicken and other poultry only account for a small portion of meat intake) and hip fracture risk in women (SMC) and men (COSM) separately. Depicted are the multivariable-adjusted hazard ratios and 95% confidence intervals (shaded) of hip fractures. The exposure was the daily frequency of meat intake and the start date January 1st 1998. Meat was modelled as a continuous exposure, using a restricted cubic spline Cox model, with three knots placed at the 10th, 50th and 90th percentile, with the reference point set to a daily frequency of two servings. The meat variable were time-updated in 2009 and 2019. The analysis was adjusted for age, height (continuous), body mass index (continuous), smoking habits (current, former, never), living alone (binary), educational level (≤9, 10-12, >12 years, other), use of calcium and vitamin D supplements (binary), cortisone use (binary), walking/cycling (never/seldom, < 20 min/d, 20-40 min/d, 40-60 min/d, 1-1.5 h/d, >1.5 h/d), leisure time physical exercise during the past year (<1 h/w, 1 h/w, 2-3 h/w, 4-5 h/w, >5 h/w), Charlson's weighted comorbidity index, energy intake, intake of fruits/vegetables and alcohol (all continuous).

Supplemental figure 2. Associations between meat intake (mainly red and processed meat, since chicken and other poultry only account for a small portion of meat intake) and hip fracture risk in the SMC cohort (women) with baseline 1987-90. Depicted are the multivariable-adjusted hazard ratios and 95% confidence intervals (shaded) of hip fractures. The exposure was the daily frequency of meat intake. Meat was modelled as a continuous exposure, using a restricted cubic spline Cox model, with three knots placed at the 10th, 50th and 90th percentile, with the reference point set to a daily frequency of two servings. The meat variable were time-updated in 1997, 2009 and 2019. The analysis was adjusted for age, height (continuous), body mass index (continuous), smoking habits (current, former, never), living alone (binary), educational level (≤9, 10-12, >12 years, other), use of calcium and vitamin D supplements (binary), cortisone use (binary), walking/cycling (never/seldom, < 20 min/d, 20-40 min/d, 40-60 min/d, 1-1.5 h/d, >1.5 h/d), leisure time physical exercise during the past year (<1 h/w, 1 h/w, 2-3 h/w, 4-5 h/w, >5 h/w), Charlson's weighted comorbidity index, energy intake, intake of fruits/vegetables and alcohol (all continuous). HR Hazard Ratio

Supplemental figure 3. Associations between meat intake (mainly red and processed meat, since chicken and other poultry only account for a small portion of meat intake) and hip fracture risk in the pooled sample of women and men. Depicted are the multivariable-adjusted hazard ratios and 95% confidence intervals (shaded) of hip fractures. The exposure was the daily frequency of meat intake with a start date of January 1st 1998. Meat was modelled as a continuous exposure, using a restricted cubic spline Cox model, with three knots placed at the 10th, 50th and 90th percentile, with the reference point set to a daily frequency of two servings. The meat variable were time-updated in 2009 and 2019. The analysis was adjusted for age, height (continuous), body mass index (continuous), smoking habits (current, former, never), living alone (binary), educational level (≤9, 10-12, >12 years, other), use of calcium and vitamin D supplements (binary), cortisone use (binary), walking/cycling (never/seldom, < 20 min/d, 20-40 min/d, 40-60 min/d, 1-1.5 h/d, >1.5 h/d), leisure time physical exercise during the past year (<1 h/w, 1 h/w, 2-3 h/w, 4-5 h/w, >5 h/w), Charlson's weighted comorbidity index, energy intake, intake of fruits/vegetables, alcohol and in addition milk, fermented milk and cheese (all continuous).

Supplemental figure 4. Associations between meat intake (mainly red and processed meat, since chicken and other poultry only account for a small portion of meat intake) and hip fracture risk in the pooled sample of women and men. Depicted are the multivariable-adjusted hazard ratios and 95% confidence intervals (shaded) of hip fractures. The exposure was the daily frequency of meat intake with a start date of January 1st 1998. Meat was modelled as a continuous exposure, using a restricted cubic spline Cox model, with three knots placed at the 10th, 50th and 90th percentile, with the reference point set to a daily frequency of two. The meat variable were time-updated in 2009 and 2019. The analysis was adjusted for age, height (continuous), body mass index (continuous), smoking habits (current, former, never), living alone (binary), educational level (≤9, 10-12, >12 years, other), use of calcium and vitamin D supplements (binary), cortisone use (binary), walking/cycling (never/seldom, < 20 min/d, 20-40 min/d, 40-60 min/d, 1-1.5 h/d, >1.5 h/d), leisure time physical exercise during the past year (<1 h/w, 1 h/w, 2-3 h/w, 4-5 h/w, >5 h/w), Charlson's weighted comorbidity index, energy intake, intake of fruits/vegetables, alcohol and in addition chicken or other poultry (all continuous).
